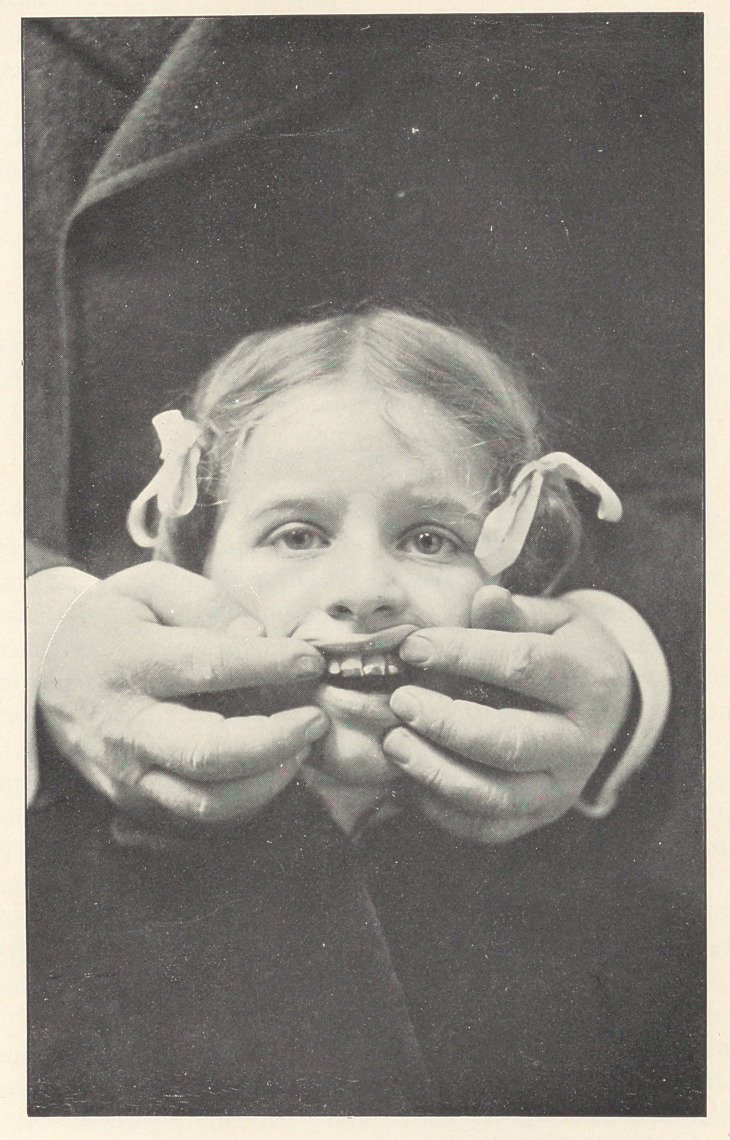# An Interesting Regulating Case

**Published:** 1901-04

**Authors:** John E. Heyke

**Affiliations:** New Haven, Conn.


### AN INTERESTING REGULATING CASE.

BY DR. JOHN E. HEYKE, NEW HAVEN, CONN.

On May 21, 1900, a girl ten years of age came to my office to have one of her central incisors regulated. The history of the case is as follows:

At four years of age the girl fell off the porch, her mouth striking some object on the ground, forcing the left central up between the alveolar plates, entirely changing the position of the permanent incisor then being developed. When the permanent tooth erupted it was found to have a horizontal position, making its appearance immediately under the floor of the nose and entering the lip just above the labial fold, not being visible at any point. Gradually it had worked its way towards the outside of the lip, and at the time mentioned nothing more than the cuticle covered it on the outside.

The impression of the mouth was taken and an appliance, consisting of gold caps and a bar crossing the space of the left central, was made and cemented in place. The tooth was then liberated from the tissues of the lip, and all the soft tissues underlying the tooth were divided.

About three weeks after the appliance was put on the right central and the two laterals became very tender, and the misplaced member very loose, so an absolute rest of two weeks was given. This occurred at three different times during the regulating period.

January 8, 1901, the tooth was in its proper place, having been turned from a horizontal to a vertical position and moved down considerably over half an inch.

The tooth is dead, not, however, from the process of regulating. The appliance used consisted of the before-mentioned gold caps and rubber bands.

The illustration represents the completion of the operation, with the retaining appliance still in place upon the cutting edges of the incisors.

## Figures and Tables

**Figure f1:**